# Performance data of multiple-precision scalar and vector BLAS operations on CPU and GPU

**DOI:** 10.1016/j.dib.2020.105506

**Published:** 2020-04-21

**Authors:** Konstantin Isupov

**Affiliations:** Department of Electronic Computing Machines, Vyatka State University, Russian Federation

**Keywords:** Multiple-precision arithmetic, Floating-point computations, Graphics processing units, CUDA, BLAS

## Abstract

Many optimized linear algebra packages support the single- and double-precision floating-point data types. However, there are a number of important applications that require a higher level of precision, up to hundreds or even thousands of digits. This article presents performance data of four dense basic linear algebra subprograms – ASUM, DOT, SCAL, and AXPY – implemented using existing extended-/multiple-precision software for conventional central processing units and CUDA compatible graphics processing units. The following open source packages are considered: MPFR, MPDECIMAL, ARPREC, MPACK, XBLAS, GARPREC, CAMPARY, CUMP, and MPRES-BLAS. The execution time of CPU and GPU implementations is measured at a fixed problem size and various levels of numeric precision. The data in this article are related to the research article entitled “Design and implementation of multiple-precision BLAS Level 1 functions for graphics processing units” [1].

Specifications table

**Table:** 

Subject	Computer Science
Specific subject area	High-Precision Computations
Type of data	Tables and CSV files
How data were acquired	Execution of the compiled source code
Data format	Raw and processed
Parameters for data collection	Hardware system: Intel Core i5-4590 (3.30 GHz, 4 Cores/4 Threads), 16 GB DDR3 RAM, NVIDIA Turing RTX 2060 GPU (1920 CUDA Cores, Compute Capability 7.5, 6 GB GDDR6 memory). Software environment: Ubuntu 19.10 (development branch), GCC compiler version 7.4.0, CUDA Toolkit 10.1.105, nvcc flags: -O3 -DNDEBUG -use_fast_math -std=c++14 -Xcompiler=-O3,-fopenmp,-ffast-math. The input data sets were composed of random numbers in the range of −1 to 1. Measurements do not include the time spent transferring data between the CPU and the GPU.
Description of data collection	Performance data were collected at a fixed problem size and various arithmetic precisions. The CPU-based codes were developed using OpenMP and executed on multiple cores. Three runs were performed for each test case. At each test run, the BLAS function under evaluation was repeated several times, and the total execution time of all iterations was measured in milliseconds. Then the average execution time for one iteration was calculated.
Data source location	Vyatka State University, Kirov, Russian Federation
Data accessibility	Processed data are with this article. Raw data are available at the Mendeley Data repository (http://dx.doi.org/10.17632/yrdh6r3sgx.2). The source code for the tests is available at GitHub (https://github.com/kisupov/mpres-blas).
Related research article	K. Isupov, V. Knyazkov, A. Kuvaev, Design and Implementation of Multiple-Precision BLAS Level 1 Functions for Graphics Processing Units, Journal of Parallel and Distributed Computing. 140 (2020) 25–36. https://doi.org/10.1016/j.jpdc.2020.02.006.

## Value of the data

• The data obtained allows comparing the efficiency (in terms of execution time) of various multiple-precision packages when performing BLAS Level 1 operations, which are the building blocks for many linear algebra algorithms.
• The data could be useful for developing GPU accelerated applications that require more precision than the standard double precision available in most existing BLAS libraries.
• They can benefit researchers dealing with scientific and engineering calculations that are sensitive to rounding errors (e.g., ill-conditioned linear systems and eigenvalue problems).
• These data can also be used to understand the impact on performance of computations with higher levels of precision performed on multicore processors and massively parallel graphics processing units.

## 1 Data description

The data presented in this paper are performance measurements of ASUM, DOT, SCAL and AXPY functions from Level 1 BLAS [2] implemented using multiple-precision software for central processing units (CPUs) and CUDA-enabled graphics processing units (GPUs). The ASUM operation computes the sum of magnitudes of the vector elements. The DOT operation computes a vector-vector dot product. The SCAL operation computes the product of a vector by a scalar. The AXPY operation computes a vector-scalar product and adds the result to a vector. All data consists of 60 CSV files (raw data) and two tables (processed data).

The raw data of the experiments are available at the Mendeley Data repository [3]. The raw data are organized in two folders named “*1. Precision from 120 to 2400 bits*” and “*2. Precision from 106 to 424 bits*”. The first folder contains the performance data of implementations using the MPFR, ARPREC, MPDECIMAL, MPACK, GARPREC, CUMP, and MPRES-BLAS packages for precisions of 120, 240, 480, 720, 960, 1200, 1440, 1680, 1920, 2160, and 2400 bits. The second folder contains the performance data of implementations using the XBLAS, CAMPARY, and MPRES-BLAS packages for precisions of 106, 212, 318, and 424 bits. Each raw file contains the results of three test runs at a fixed operation size of 1,000,000. For each test run, the BLAS function was repeated ten times, and the raw file presents the total execution time of ten iterations (in milliseconds).

The processed data are reported in Tables 1 and 2. Table 1 presents the average execution time of the MPFR, ARPREC, MPDECIMAL, MPACK, GARPREC, CUMP, and MPRES-BLAS packages with precisions from 120 to 2400 bits. Table 2 reports the average time of the XBLAS, CAMPARY, and MPRES-BLAS packages with precisions of 106, 212, 318, and 424 bits. The tables allow evaluating the benefits of using GPUs to perform computation with extended/multiple precision.

**Table 1:** Average execution time of multiple-precision BLAS Level 1 operations based on MPFR, ARPREC, MPDECIMAL, MPACK, GARPREC, CUMP, and MPRES-BLAS. Measurements are in milliseconds.

Precision, bits	Intel Core i5 4590				NVIDIA Turing RTX 2060		
	MPFR	ARPREC	MPDECIMAL	MPACK	GARPREC	CUMP	MPRES-BLAS
Sum of absolute values (ASUM)							
120	7.05	60.31	55.12	114.03	2.78	4.43	0.79
240	9.30	62.82	55.63	129.57	3.79	5.23	1.27
480	9.75	66.30	57.35	128.37	7.38	6.89	2.98
720	11.25	80.50	57.01	139.05	13.63	9.96	3.93
960	13.31	91.86	58.07	142.67	15.16	12.94	5.95
1200	14.20	91.54	58.39	153.44	18.30	16.60	6.46
1440	15.22	93.83	60.81	154.51	21.49	19.94	9.29
1680	17.05	98.35	60.76	164.56	26.85	23.47	8.96
1920	19.31	108.70	59.72	174.48	29.65	25.57	11.91
2160	22.70	109.41	59.04	197.77	32.23	29.41	11.20
2400	22.98	117.13	61.57	201.50	35.51	32.13	16.07

Dot product of two vectors (DOT)							
120	14.16	100.03	56.93	29.51	9.70	3.84	1.93
240	16.34	112.55	62.62	42.08	13.01	5.01	2.93
480	19.11	170.23	58.53	59.93	31.95	8.08	5.84
720	24.82	246.81	62.19	78.84	50.01	12.86	8.43
960	27.69	353.26	71.95	99.19	85.07	16.88	10.48
1200	32.26	430.39	92.62	134.25	115.05	23.05	12.51
1440	36.18	565.67	104.22	66.61	156.96	29.87	16.05
1680	41.95	741.57	131.85	74.48	222.28	37.93	16.67
1920	47.10	938.14	157.48	89.72	278.08	44.36	20.82
2160	53.79	1096.66	204.60	101.37	328.02	53.30	21.49
2400	59.14	1338.25	221.97	109.38	402.19	62.67	25.60

Vector-scalar product (SCAL)							
120	6.93	43.12	27.52	92.29	6.83	0.61	0.79
240	11.80	58.52	28.42	106.25	9.62	0.95	1.10
480	23.64	107.11	28.07	115.61	25.51	1.93	1.88
720	40.78	173.31	32.08	134.30	42.21	3.72	3.38
960	66.72	289.82	40.59	165.11	74.27	5.53	3.22
1200	92.92	386.84	48.62	197.50	102.64	8.35	5.10
1440	26.28	514.54	65.99	235.13	142.76	11.56	4.58
1680	30.39	732.00	90.03	284.39	203.65	14.99	6.57
1920	33.47	901.76	111.28	332.91	256.73	18.73	5.82
2160	38.86	1051.37	136.83	388.47	306.08	22.96	8.12
2400	44.04	1236.25	162.87	468.98	376.58	27.79	7.56

Constant times a vector plus a vector (AXPY)							
120	11.56	76.79	59.40	30.53	9.11	1.30	2.23
240	15.13	92.98	61.76	37.14	12.33	2.34	2.99
480	18.05	166.53	63.64	42.82	26.92	4.74	4.63
720	24.91	253.53	69.85	57.69	46.24	7.98	7.27
960	28.22	382.67	79.03	61.34	79.77	10.95	7.52
1200	32.82	503.74	102.08	75.93	107.60	15.47	10.44
1440	37.57	661.71	116.52	93.61	150.13	20.12	10.50
1680	42.32	885.10	135.75	108.75	209.87	25.28	13.02
1920	49.09	1091.15	166.22	129.85	268.98	30.04	13.12
2160	52.97	1285.11	192.59	151.76	316.18	35.94	16.27
2400	62.06	1466.24	218.83	167.63	389.25	42.49	16.44

**Table 2:** Average execution time of extended-precision BLAS Level 1 operations based on XBLAS, CAMPARY, and MPRES-BLAS. A value of “N/A” indicates that the corresponding operation or precision is not supported. Measurements are in milliseconds.

Operation	Package	Precision, bits			
		106	212	318	424
ASUM	XBLAS (Intel Core i5, 1 thread)	6.13	N/A	N/A	N/A
	CAMPARY (NVIDIA Turing RTX 2060)	0.29	1.75	3.33	5.27
	MPRES-BLAS (NVIDIA Turing RTX 2060)	0.78	1.31	1.62	3.00

DOT	XBLAS (Intel Core i5, 1 thread)	9.73	N/A	N/A	N/A
	CAMPARY (NVIDIA Turing RTX 2060)	0.35	4.42	11.17	22.19
	MPRES-BLAS (NVIDIA Turing RTX 2060)	1.95	2.94	4.01	5.89

SCAL	XBLAS (Intel Core i5, 1 thread)	N/A	N/A	N/A	N/A
	CAMPARY (NVIDIA Turing RTX 2060)	0.13	2.65	7.59	16.73
	MPRES-BLAS (NVIDIA Turing RTX 2060)	0.78	1.10	1.89	1.86

AXPY	XBLAS (Intel Core i5, 1 thread)	5.33	N/A	N/A	N/A
	CAMPARY (NVIDIA Turing RTX 2060)	0.28	3.94	10.04	18.48
	MPRES-BLAS (NVIDIA Turing RTX 2060)	2.24	2.98	4.31	4.62

## 2 Experimental design, materials, and methods

All the experiments were carried out at a fixed operation size of 1,000,000. The input vectors were composed of randomly generated floating-point numbers in the range [−1; 1]. In order to generate uniformly distributed random significands, we used the *mpz_urandomb* function from the GNU MP Bignum Library (https://gmplib.org/). Measurements do not include the time spent transferring data between the CPU and the GPU. We have also excluded the time of converting data into internal multiple-precision representations.

The function *clock_gettime* was used to measure the execution times of CPU implementations. For GPU implementations, the execution times were measured using CUDA Events. In order to reduce the impact of noise, no other applications were launched during the test execution, and the GUI was not used. Three runs were performed for each test case. At each test run, the BLAS function under evaluation was repeated ten times, and the total execution time of all iterations was measured.

A summary of the experimental setup is given in Table 3. Table 4 contains a brief description of the considered multiple-precision software.

**Table 3:** Experimental setup summary.

Host	GPU
Intel Core i5 4590	NVIDIA Turing RTX 2060 (1.68 GHz)
4 Cores / 4 Threads	1920 CUDA Cores
16 GB DDR3 RAM	Compute Capability 7.5
Ubuntu 19.10 (development branch)	6 GB GDDR6
GCC 7.4.0	CUDA Toolkit 10.1.105
Compile options: -O3,-fopenmp,-ffast-math	Compile options: -O3 -DNDEBUG -use_fast_math -std=c++14

**Table 4:** Multiple-precision software.

Package	Target platform	Description	URL	How was compiled / installed
MPFR [4]	CPU	A C library for multiple-precision floating-point computations with correct rounding	https://www.mpfr.org	sudo apt-get install libmpfr-dev
ARPREC [5]	CPU	An arbitrary precision package for Fortran and C++	https://www.davidhbailey.com/dhbsoftware	Compiled by GCC 7.4.0 using the provided scripts
MPDECIMAL (libmpdec) [6]	CPU	A package for correctly-rounded arbitrary precision decimal floating point	https://www.bytereef.org/mpdecimal	Compiled by GCC 7.4.0 using the provided scripts
MPACK [7]	CPU	Multiple-precision versions of BLAS and LAPACK	http://mplapack.sourceforge.net	Compiled by GCC 7.4.0 using the provided scripts
XBLAS [8]	CPU	A reference implementation of extended and mixed precision BLAS routines	https://www.netlib.org/xblas	Compiled by G++ 7.4.0 using the provided scripts
GARPREC [9]	GPU	A port of the ARPREC package for CUDA-enabled GPUs	https://code.google.com/archive/p/gpuprec/downloads	Compiled by nvcc 10.1 as part of the test executable
CAMPARY [10]	CPU and GPU	A multiple-precision library that uses floating-point expansions to represent extended precision numbers	http://homepages.laas.fr/mmjoldes/campary	Compiled by nvcc 10.1 as part of the test executable
CUMP [11]	GPU	A library for arbitrary precision arithmetic on CUDA based on the GNU MP Bignum Library	https://github.com/skystar0227/CUMP	Compiled by nvcc 10.1 using the provided scripts
MPRES-BLAS [1]	GPU	Multiple-precision GPU accelerated BLAS functions based on residue number system	https://github.com/kisupov/mpres-blas	Compiled by nvcc 10.1 as part of the test executable

Using arithmetic operations from MPFR, ARPERC, MPDECIMAL, GARPREC, CUMP and CAMPARY, we have implemented multiple-precision ASUM, DOT, SCAL, and AXPY for CPU and GPU. The CPU-based codes were developed using OpenMP and executed in parallel with 4 threads on 4 physical cores.

For MPACK, we used the *mpreal* data type and the *Rasum, Rdot, Rscal*, and *Raxpy* routines, which are based on MPFR C++ (http://www.holoborodko.com/pavel/mpfr/). Note that only the *Rdot* and *Raxpy* routines support multi-threaded calculations, and these routines were performed with 4 OpenMP threads, whereas *Rasum* and *Rscal* were performed with a single thread.

For XBLAS, the double-double precision routines *BLAS_dsum_x, BLAS_ddot_x*, and *BLAS_dwaxpby_x* were evaluated, which provide 106 bits of internal precision. Since XBLAS does not support parallel computation, these routines were executed with a single thread. Note that the *BLAS_dsum_x* routine computes the sum of the vector elements, not the sum of absolute values of the vector elements. Furthermore, XBLAS does not implement the SCAL operation.

In the case of MPRES-BLAS, we used the routines *mpasum, mpdot, mpscal*, and *mpaxpy*. These routines are implemented as host functions that invoke GPU kernels. Each routine has a set of template parameters that specify the kernel execution configurations. These parameters are described in Table 5. Table 6 shows the kernel execution configurations used in the experiments. Among the various configurations considered, these configurations provide better performance on the machine employed in the experiments.

**Table 5:** Template parameters of the MPRES-BLAS routines; for details, see [1].

Routine	Parameter	Description
mpasum	gridDim1	The number of blocks for parallel summation
	blockDim1	The number of threads per block for parallel summation
mpdot	gridDim1	The number of blocks for computing the signs, exponents, RNS interval evaluations, and for rounding the result in vector-vector multiplication
	blockDim1	The number of threads per block for computing the signs, exponents, RNS interval evaluations, and for rounding the result in vector-vector multiplication
	gridDim2	The number of blocks for computing the digits (residues) of multiple-precision significands in vector-vector multiplication
	gridDim3	The number of blocks for reducing the vector of products
	blockDim3	The number of threads per block for reducing the vector of products
mpscal, mpaxpy	gridDim1	The number of blocks for computing the signs, exponents, RNS interval evaluations, and for rounding the result
	blockDim1	The number of threads per block for computing the signs, exponents, RNS interval evaluations, and also for rounding the result
	gridDim2	The number of blocks for computing the digits (residues) of multiple-precision significands

**Table 6:** MPRES-BLAS execution configurations used in the experiments.

Precision, bits	mpasum		mpdot					mpscal, mpaxpy		
	gridDim1	blockDim1	gridDim1	blockDim1	gridDim2	gridDim3	blockDim3	gridDim1	blockDim1	gridDim2
120	256	128	512	128	8192	256	128	512	128	8192
240	256	128	512	128	8192	256	128	512	128	8192
480	256	128	512	128	8192	256	128	512	128	8192
720	256	64	512	128	8192	256	64	512	128	8192
960	256	64	512	128	8192	256	64	512	128	8192
1200	256	32	512	128	8192	256	32	512	128	8192
1440	512	64	512	128	8192	512	64	512	128	8192
1680	512	64	512	128	8192	512	64	512	128	8192
1920	512	32	512	128	8192	512	32	512	128	8192
2160	256	32	512	128	8192	256	32	512	128	8192
2400	512	32	512	128	8192	512	32	512	128	8192

106	256	128	512	128	8192	256	128	512	128	8192
212	256	128	512	128	8192	256	128	512	128	8192
318	256	128	512	128	8192	256	128	512	128	8192
424	256	128	512	128	8192	256	128	512	128	8192
